# 4-Chloro-*N*-[*N*-(6-methyl-2-pyrid­yl)car­bamo­thio­yl]benzamide

**DOI:** 10.1107/S1600536808041123

**Published:** 2008-12-10

**Authors:** Gün Binzet, Fatih Mehmet Emen, Ulrich Flörke, Tuncay Yeşilkaynak, Nevzat Külcü, Hakan Arslan

**Affiliations:** a Department of Chemistry, Faculty of Arts and Science, Mersin University, 33343 Mersin, Turkey; b Department of Chemistry, University of Paderborn, 33098 Paderborn, Germany; c Department of Natural Sciences, Fayetteville State University, Fayetteville, NC, 28301, USA; d Department of Chemistry, Faculty of Pharmacy, Mersin University, 33169 Mersin, Turkey

## Abstract

In the title compound, C_14_H_12_ClN_3_OS, the short exocyclic N—C bond lengths indicate resonance in the thiourea part of the mol­ecule. The title compound is stabilized by an intra­molecular N—H⋯N hydrogen bond, which results in the formation of a six-membered ring. In addition, it shows a synperiplanar conformation between the thio­carbonyl group and the pyridine group. Inter­molecular N—H⋯S and C—H⋯O inter­actions are also present.

## Related literature

For the synthesis, see: Mansuroğlu *et al.* (2008 ▶); Arslan *et al.* (2003*a* ▶,*b* ▶); Binzet *et al.* (2006 ▶). For general background, see: Arslan *et al.* (2006*a* ▶,*b* ▶, 2007 ▶); Kemp *et al.* (1997 ▶); Koch *et al.* (1995 ▶); Nencki (1873 ▶); Özpozan *et al.* (2000 ▶). For related compounds, see: Arslan *et al.* (2003*a* ▶, 2006*b* ▶, 2007 ▶); Dong *et al.* (2008 ▶); Duque *et al.* (2008 ▶); Tutughamiarso & Bolte (2007 ▶); Yue *et al.* (2008 ▶); Yusof *et al.* (2008*a* ▶,*b* ▶); Xian (2008 ▶); Thiam *et al.* (2008 ▶); Binzet *et al.* (2006 ▶); Uğur *et al.* (2006 ▶).
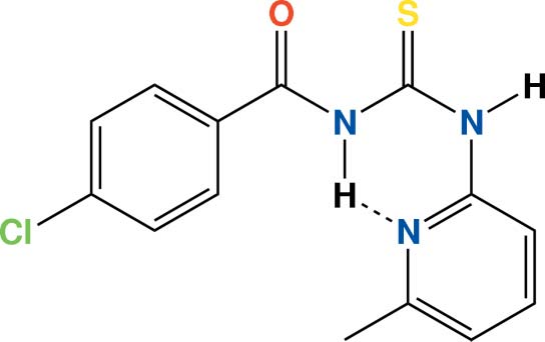


## Experimental

### 

#### Crystal data

C_14_H_12_ClN_3_OS
*M* *_r_* = 305.78
Triclinic, 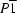

*a* = 8.255 (3) Å
*b* = 9.030 (3) Å
*c* = 9.957 (3) Å
α = 80.810 (7)°
β = 66.552 (7)°
γ = 87.269 (7)°
*V* = 672.1 (4) Å^3^
*Z* = 2
Mo *K*α radiation
μ = 0.44 mm^−1^
*T* = 120 (2) K
0.20 × 0.14 × 0.10 mm

#### Data collection

Bruker SMART CCD area-detector diffractometer
Absorption correction: multi-scan (*SADABS*; Bruker, 2002 ▶) *T*_min_ = 0.918, *T*_max_ = 0.958
3651 measured reflections
2609 independent reflections
1492 reflections with *I* > 2σ(*I*)
*R*_int_ = 0.100

#### Refinement

*R*[*F* ^2^ > 2σ(*F* ^2^)] = 0.077
*wR*(*F* ^2^) = 0.266
*S* = 0.98
2609 reflections
182 parameters
H-atom parameters constrained
Δρ_max_ = 0.81 e Å^−3^
Δρ_min_ = −0.50 e Å^−3^

### 

Data collection: *SMART* (Bruker, 2002 ▶); cell refinement: *SAINT* (Bruker, 2002 ▶); data reduction: *SAINT*; program(s) used to solve structure: *SHELXTL* (Sheldrick, 2008 ▶); program(s) used to refine structure: *SHELXTL*; molecular graphics: *SHELXTL*; software used to prepare material for publication: *SHELXTL*.

## Supplementary Material

Crystal structure: contains datablocks global, I. DOI: 10.1107/S1600536808041123/hg2444sup1.cif

Structure factors: contains datablocks I. DOI: 10.1107/S1600536808041123/hg2444Isup2.hkl

Additional supplementary materials:  crystallographic information; 3D view; checkCIF report

## Figures and Tables

**Table 1:** Hydrogen-bond geometry (Å, °)

*D*—H⋯*A*	*D*—H	H⋯*A*	*D*⋯*A*	*D*—H⋯*A*
C12—H12*A*⋯O1^i^	0.95	2.42	3.303 (7)	154
N2—H2*B*⋯S1^ii^	0.88	2.61	3.464 (5)	165
N1—H1*B*⋯N3	0.88	1.90	2.651 (7)	142

Symmetry codes: (i) 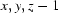; (ii) 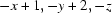.

**Fig. 1.**
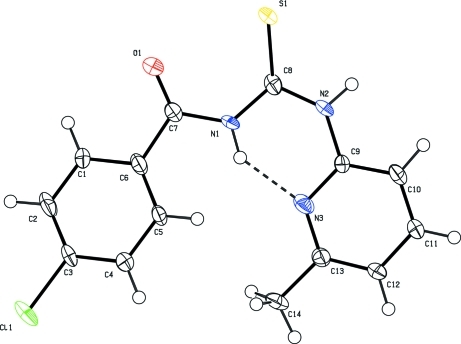
The molecular structure of the title compound, showing the atom-numbering scheme and displacement ellipsoids drawn at the 50% probability level.

**Fig. 2.**
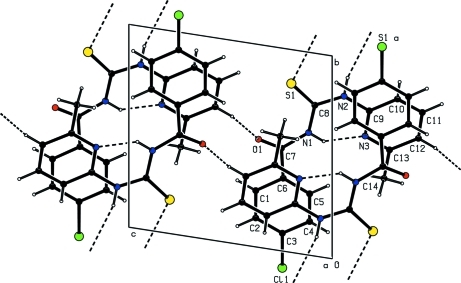
Packing diagram for (I) viewed along a-direction. Hydrogen bonds shown as dotted lines.

## supplementary crystallographic information

### Comment

Thiourea derivatives, first synthesized by Nencki, 1873, are very flexible ligands and able to coordinate to a range of metal centers as neutral ligands, monoanions or dianions (Mansuroğlu *et al.*, 2008; Arslan *et al.*, 2003*b*, 2006*a*; Binzet *et al.*, 2006; Kemp *et al.*, 1997; Koch *et al.*, 1995). In addition, the oxygen, nitrogen and sulfur donors provide a multitude of bonding possibilities. The coordination chemistry of substituted thioureas has led to some interesting practical applications such as liquid–liquid extraction, pre-concentration and highly efficient chromatographic separation (Kemp *et al.*, 1997; Koch *et al.*, 1995).

Our team focused on the synthesis, characterization, crystal structure, thermal behavior and antimicrobial activity of new thiourea derivatives (Mansuroğlu *et al.*, 2008; Arslan *et al.*, 2003*a*, 2003*b*, 2006*a*, 2006*b*, 2007; Uğur *et al.*, 2006; Özpozan *et al.*, 2000). In this article, we report the preparation and characterization of a novel thiourea compound, 4-chloro-*N*-(6-methylpyridin-2-yl-carbamothioyl)benzamide (I), and its crystal structure. The title compound was purified by re-crystallization from ethanol:dichloromethane mixture (1:2) and characterized by elemental analysis. The analytical data is consistent with the proposed structure given in Scheme 1.

The molecular structure and packing diagram are depicted in Figure 1 and 2, respectively. The bond lengths and angles in the thiourea moiety are typical for thiourea derivatives; the C8—S1 and C7—O1 bonds both show a typical double-bond character with 1.655 (5) and 1.203 (6) Å, respectively. The short bond lengths of the N1—C7, 1.395 (7); N1—C8, 1.376 (6) and N2—C8, 1.364 (6) Å bonds indicate partial double bond character. These results can be explained by the existence of resonance in this part of the molecule. The other C—N bond length is within the expected range.

A lot of substitute benzoylthiourea derivatives have *cis*-*trans* configurations (Yusof, *et al.*, 2008*a*, 2008*b*; Thiam, *et al.*, 2008; Xian, 2008; Dong, *et al.*, 2008; Duque, *et al.*, 2008). However, the title compound shows an intramolecular N—H···N hydrogen bond (Table 1) which results in the formation of a six membered ring (N3—C9—N2—C8—N1—H1) and leads to a *syn*-periplanar conformation between the thiocarbonyl group carbon atom and the pyridine group nitrogen atom (Tutughamiarso & Bolte, 2007; Yue *et al.*, 2008). The torsion angles in this region, C8—N2—C9—N3, 14.9 (9)° and C9—N2—C8—N1, -7.2 (8)° confirm this conformation. This formation forces the two amide hydrogen atoms to the opposite direction.

The carbonyl and thiocarbonyl part is essentially planar, as reflected by the torsional angles O1—C7—N1—C8, C7—N1—C8—S1 and C7—N1—C8—N2 of 1.7 (9), 0.3 (8) and 179.3 (5) °, respectively. O1, C7, N1, C8 and S1 fragment is also planar (maximum and mean deviations are 0.010 and 0.005 Å, respectively).

The crystal packing is shown in Fig. 2. There are two intermolecular, N—H···S and C—H···O, hydrogen bonds which connect molecules in chains parallel to [100].

### Experimental

The compound was prepared with a procedure similar to that reported in the literature (Arslan *et al.*, 2003*b*, 2006*a*). A solution of 4-chloro-benzoyl chloride (0.01 mol) in acetone (50 cm^3^) was added dropwise to a suspension of potassium thiocyanate (0.01 mol) in acetone (30 cm^3^). The reaction mixture was heated under reflux for 30 min, and then cooled to room temperature. A solution of 6-methylpyridin-2-amine (0.01 mol) in acetone (10 cm^3^) was added and the resulting mixture was stirred for 2 h. Hydrochloric acid (0.1 N, 300 cm^3^) was added to the solution, which was then filtered. The solid product was washed with water and purifed by recrystalization from an ethanol:dichloromethane mixture (1:2). Anal. Calcd. for C_14_H_12_ClN_3_OS: C, 55.0; H, 4.0; N, 13.7. Found: C, 55.1; H, 3.9; N, 13.7%.

### Crystal data

**Table:**

C_14_H_12_ClN_3_OS	*Z* = 2
*M**_r_* = 305.78	*F*(000) = 316
Triclinic, *P*1	*D*_x_ = 1.511 Mg m^−^^3^
Hall symbol: -P 1	Mo *K*α radiation, λ = 0.71073 Å
*a* = 8.255 (3) Å	Cell parameters from 716 reflections
*b* = 9.030 (3) Å	θ = 2.3–26.3°
*c* = 9.957 (3) Å	µ = 0.44 mm^−^^1^
α = 80.810 (7)°	*T* = 120 K
β = 66.552 (7)°	Prism, yellow
γ = 87.269 (7)°	0.20 × 0.14 × 0.10 mm
*V* = 672.1 (4) Å^3^	

### Data collection

**Table:**

Bruker SMART CCD area-detector diffractometer	2609 independent reflections
Radiation source: sealed tube	1492 reflections with *I* > 2σ(*I*)
graphite	*R*_int_ = 0.100
φ and ω scans	θ_max_ = 26.4°, θ_min_ = 2.3°
Absorption correction: multi-scan (*SADABS*; Bruker, 2002)	*h* = −10→10
*T*_min_ = 0.918, *T*_max_ = 0.958	*k* = −11→5
3651 measured reflections	*l* = −12→10

### Refinement

**Table:**

Refinement on *F*^2^	Primary atom site location: structure-invariant direct methods
Least-squares matrix: full	Secondary atom site location: difference Fourier map
*R*[*F*^2^ > 2σ(*F*^2^)] = 0.077	Hydrogen site location: inferred from neighbouring sites
*wR*(*F*^2^) = 0.266	H-atom parameters constrained
*S* = 0.98	*w* = 1/[σ^2^(*F*_o_^2^) + (0.1601*P*)^2^] where *P* = (*F*_o_^2^ + 2*F*_c_^2^)/3
2609 reflections	(Δ/σ)_max_ < 0.001
182 parameters	Δρ_max_ = 0.81 e Å^−^^3^
0 restraints	Δρ_min_ = −0.50 e Å^−^^3^

### Special details

**Table:**

Geometry. All e.s.d.'s (except the e.s.d. in the dihedral angle between two l.s. planes) are estimated using the full covariance matrix. The cell e.s.d.'s are taken into account individually in the estimation of e.s.d.'s in distances, angles and torsion angles; correlations between e.s.d.'s in cell parameters are only used when they are defined by crystal symmetry. An approximate (isotropic) treatment of cell e.s.d.'s is used for estimating e.s.d.'s involving l.s. planes.
Refinement. Refinement of *F*^2^ against ALL reflections. The weighted *R*-factor *wR* and goodness of fit *S* are based on *F*^2^, conventional *R*-factors *R* are based on *F*, with *F* set to zero for negative *F*^2^. The threshold expression of *F*^2^ > σ(*F*^2^) is used only for calculating *R*-factors(gt) *etc*. and is not relevant to the choice of reflections for refinement. *R*-factors based on *F*^2^ are statistically about twice as large as those based on *F*, and *R*- factors based on ALL data will be even larger.

### Fractional atomic coordinates and isotropic or equivalent isotropic displacement parameters (Å^2^)

**Table:**

	*x*	*y*	*z*	*U*_iso_*/*U*_eq_	
Cl1	−0.0760 (2)	−0.08311 (16)	0.24521 (19)	0.0469 (5)	
S1	0.4068 (2)	0.83271 (15)	0.20068 (15)	0.0316 (5)	
O1	0.2334 (6)	0.5331 (4)	0.3623 (4)	0.0370 (10)	
N1	0.2869 (6)	0.6040 (5)	0.1158 (5)	0.0296 (11)	
H1B	0.2730	0.5725	0.0418	0.036*	
N2	0.4090 (6)	0.8104 (5)	−0.0583 (5)	0.0282 (11)	
H2B	0.4462	0.9040	−0.0760	0.034*	
N3	0.3162 (6)	0.6306 (5)	−0.1620 (5)	0.0253 (10)	
C1	0.0432 (8)	0.2777 (6)	0.3757 (6)	0.0347 (14)	
H1A	0.0183	0.3141	0.4662	0.042*	
C2	−0.0314 (8)	0.1427 (6)	0.3760 (7)	0.0394 (15)	
H2A	−0.1122	0.0888	0.4661	0.047*	
C3	0.0122 (8)	0.0878 (6)	0.2450 (6)	0.0322 (13)	
C4	0.1259 (8)	0.1664 (6)	0.1110 (6)	0.0365 (15)	
H4A	0.1537	0.1280	0.0211	0.044*	
C5	0.1987 (7)	0.3030 (6)	0.1107 (6)	0.0264 (12)	
H5A	0.2783	0.3572	0.0201	0.032*	
C6	0.1558 (7)	0.3597 (5)	0.2407 (6)	0.0258 (12)	
C7	0.2278 (7)	0.5061 (6)	0.2497 (6)	0.0272 (12)	
C8	0.3644 (6)	0.7438 (5)	0.0850 (6)	0.0208 (11)	
C9	0.4067 (7)	0.7576 (6)	−0.1828 (6)	0.0261 (12)	
C10	0.5010 (7)	0.8375 (6)	−0.3213 (6)	0.0291 (13)	
H10A	0.5701	0.9232	−0.3321	0.035*	
C11	0.4925 (8)	0.7902 (6)	−0.4435 (6)	0.0316 (13)	
H11A	0.5503	0.8463	−0.5394	0.038*	
C12	0.3987 (7)	0.6598 (6)	−0.4245 (6)	0.0287 (13)	
H12A	0.3913	0.6251	−0.5071	0.034*	
C13	0.3156 (7)	0.5806 (6)	−0.2823 (6)	0.0229 (12)	
C14	0.2159 (8)	0.4360 (6)	−0.2553 (7)	0.0322 (13)	
H14A	0.2846	0.3515	−0.2327	0.048*	
H14B	0.1965	0.4249	−0.3441	0.048*	
H14C	0.1018	0.4375	−0.1715	0.048*	

### Atomic displacement parameters (Å^2^)

**Table:**

	*U*^11^	*U*^22^	*U*^33^	*U*^12^	*U*^13^	*U*^23^
Cl1	0.0577 (11)	0.0250 (8)	0.0548 (11)	−0.0248 (7)	−0.0183 (9)	−0.0001 (7)
S1	0.0473 (10)	0.0223 (7)	0.0274 (8)	−0.0173 (6)	−0.0164 (7)	−0.0004 (6)
O1	0.053 (3)	0.030 (2)	0.029 (2)	−0.0182 (19)	−0.017 (2)	−0.0027 (17)
N1	0.043 (3)	0.023 (2)	0.022 (2)	−0.019 (2)	−0.009 (2)	−0.0055 (19)
N2	0.038 (3)	0.020 (2)	0.027 (2)	−0.0162 (19)	−0.013 (2)	−0.0017 (18)
N3	0.024 (2)	0.022 (2)	0.034 (3)	−0.0071 (18)	−0.015 (2)	−0.0014 (19)
C1	0.059 (4)	0.022 (3)	0.019 (3)	−0.015 (3)	−0.012 (3)	0.004 (2)
C2	0.045 (4)	0.027 (3)	0.034 (3)	−0.022 (3)	−0.003 (3)	0.003 (3)
C3	0.040 (3)	0.022 (3)	0.029 (3)	−0.019 (2)	−0.009 (3)	0.005 (2)
C4	0.064 (4)	0.021 (3)	0.024 (3)	−0.014 (3)	−0.017 (3)	0.001 (2)
C5	0.033 (3)	0.020 (3)	0.026 (3)	−0.013 (2)	−0.015 (2)	0.007 (2)
C6	0.030 (3)	0.017 (3)	0.030 (3)	−0.008 (2)	−0.013 (3)	0.004 (2)
C7	0.035 (3)	0.020 (3)	0.027 (3)	−0.009 (2)	−0.015 (3)	0.002 (2)
C8	0.020 (3)	0.017 (2)	0.029 (3)	0.000 (2)	−0.016 (2)	0.004 (2)
C9	0.043 (3)	0.017 (3)	0.020 (3)	−0.009 (2)	−0.016 (3)	0.004 (2)
C10	0.036 (3)	0.020 (3)	0.032 (3)	−0.013 (2)	−0.015 (3)	0.003 (2)
C11	0.047 (4)	0.020 (3)	0.024 (3)	−0.013 (2)	−0.011 (3)	0.001 (2)
C12	0.036 (3)	0.025 (3)	0.024 (3)	−0.014 (2)	−0.010 (2)	−0.004 (2)
C13	0.027 (3)	0.021 (3)	0.026 (3)	−0.004 (2)	−0.016 (2)	0.001 (2)
C14	0.042 (3)	0.024 (3)	0.038 (3)	−0.011 (2)	−0.022 (3)	−0.006 (2)

### Geometric parameters (Å, °)

**Table:**

Cl1—C3	1.737 (5)	C4—C5	1.396 (7)
S1—C8	1.655 (5)	C4—H4A	0.9500
O1—C7	1.203 (6)	C5—C6	1.376 (7)
N1—C8	1.376 (6)	C5—H5A	0.9500
N1—C7	1.395 (7)	C6—C7	1.504 (7)
N1—H1B	0.8800	C9—C10	1.382 (7)
N2—C8	1.364 (6)	C10—C11	1.379 (7)
N2—C9	1.405 (6)	C10—H10A	0.9500
N2—H2B	0.8800	C11—C12	1.386 (7)
N3—C9	1.341 (6)	C11—H11A	0.9500
N3—C13	1.347 (7)	C12—C13	1.392 (7)
C1—C2	1.391 (7)	C12—H12A	0.9500
C1—C6	1.405 (7)	C13—C14	1.506 (7)
C1—H1A	0.9500	C14—H14A	0.9800
C2—C3	1.375 (8)	C14—H14B	0.9800
C2—H2A	0.9500	C14—H14C	0.9800
C3—C4	1.390 (8)		
			
C8—N1—C7	128.0 (4)	O1—C7—C6	122.3 (5)
C8—N1—H1B	116.0	N1—C7—C6	113.2 (4)
C7—N1—H1B	116.0	N2—C8—N1	113.5 (4)
C8—N2—C9	132.1 (4)	N2—C8—S1	119.5 (4)
C8—N2—H2B	113.9	N1—C8—S1	127.1 (4)
C9—N2—H2B	113.9	N3—C9—C10	123.1 (5)
C9—N3—C13	118.0 (5)	N3—C9—N2	118.5 (5)
C2—C1—C6	119.5 (5)	C10—C9—N2	118.3 (5)
C2—C1—H1A	120.2	C11—C10—C9	118.6 (5)
C6—C1—H1A	120.2	C11—C10—H10A	120.7
C3—C2—C1	119.7 (5)	C9—C10—H10A	120.7
C3—C2—H2A	120.2	C10—C11—C12	119.2 (5)
C1—C2—H2A	120.2	C10—C11—H11A	120.4
C2—C3—C4	121.4 (5)	C12—C11—H11A	120.4
C2—C3—Cl1	119.9 (4)	C11—C12—C13	118.8 (5)
C4—C3—Cl1	118.7 (4)	C11—C12—H12A	120.6
C3—C4—C5	118.8 (5)	C13—C12—H12A	120.6
C3—C4—H4A	120.6	N3—C13—C12	122.1 (5)
C5—C4—H4A	120.6	N3—C13—C14	116.7 (5)
C6—C5—C4	120.5 (5)	C12—C13—C14	121.2 (5)
C6—C5—H5A	119.8	C13—C14—H14A	109.5
C4—C5—H5A	119.8	C13—C14—H14B	109.5
C5—C6—C1	120.1 (5)	H14A—C14—H14B	109.5
C5—C6—C7	123.7 (5)	C13—C14—H14C	109.5
C1—C6—C7	116.2 (5)	H14A—C14—H14C	109.5
O1—C7—N1	124.4 (5)	H14B—C14—H14C	109.5
			
C6—C1—C2—C3	3.0 (10)	C9—N2—C8—N1	−7.2 (8)
C1—C2—C3—C4	−1.9 (10)	C9—N2—C8—S1	172.4 (5)
C1—C2—C3—Cl1	178.3 (5)	C7—N1—C8—N2	179.3 (5)
C2—C3—C4—C5	0.9 (10)	C7—N1—C8—S1	−0.3 (8)
Cl1—C3—C4—C5	−179.3 (4)	C13—N3—C9—C10	−1.1 (8)
C3—C4—C5—C6	−1.2 (9)	C13—N3—C9—N2	−179.7 (4)
C4—C5—C6—C1	2.3 (9)	C8—N2—C9—N3	14.9 (9)
C4—C5—C6—C7	−179.8 (5)	C8—N2—C9—C10	−163.8 (5)
C2—C1—C6—C5	−3.3 (9)	N3—C9—C10—C11	4.4 (9)
C2—C1—C6—C7	178.7 (5)	N2—C9—C10—C11	−177.0 (5)
C8—N1—C7—O1	1.7 (9)	C9—C10—C11—C12	−3.7 (8)
C8—N1—C7—C6	−177.9 (5)	C10—C11—C12—C13	0.1 (9)
C5—C6—C7—O1	−157.3 (6)	C9—N3—C13—C12	−2.8 (8)
C1—C6—C7—O1	20.7 (8)	C9—N3—C13—C14	178.7 (5)
C5—C6—C7—N1	22.3 (8)	C11—C12—C13—N3	3.3 (8)
C1—C6—C7—N1	−159.7 (5)	C11—C12—C13—C14	−178.3 (5)

### Hydrogen-bond geometry (Å, °)

**Table:**

*D*—H···*A*	*D*—H	H···*A*	*D*···*A*	*D*—H···*A*
C12—H12A···O1^i^	0.95	2.42	3.303 (7)	154
N2—H2B···S1^ii^	0.88	2.61	3.464 (5)	165
N1—H1B···N3	0.88	1.90	2.651 (7)	142

Symmetry codes: (i) *x*, *y*, *z*−1; (ii) −*x*+1, −*y*+2, −*z*.

## References

[bb1] Arslan, H., Flörke, U. & Külcü, N. (2003*a*). *J. Chem. Crystallogr.* **33**, 919–924.

[bb2] Arslan, H., Flörke, U. & Külcü, N. (2007). *Spectrochim. Acta A*, **67**, 936–943. 10.1016/j.saa.2006.09.011 17049302

[bb3] Arslan, H., Flörke, U., Külcü, N. & Emen, M. F. (2006*a*). *J. Coord. Chem.* **59**, 223–228.

[bb4] Arslan, H., Külcü, N. & Flörke, U. (2003*b*). *Transition Met. Chem.* **28**, 816–819.

[bb5] Arslan, H., Külcü, N. & Flörke, U. (2006*b*). *Spectrochim. Acta A*, **64**, 1065–1071. 10.1016/j.saa.2005.09.016 16455292

[bb6] Binzet, G., Arslan, H., Flörke, U., Külcü, N. & Duran, N. (2006). *J. Coord. Chem.* **59**, 1395–1406.

[bb7] Bruker (2002). *SMART*, *SAINT* and *SADABS* Bruker AXS Inc., Madison, Wisconsin, USA.

[bb8] Dong, W.-K., Yan, H.-B., Chai, L.-Q., Lv, Z.-W. & Zhao, C.-Y. (2008). *Acta Cryst.* E**64**, o1097. 10.1107/S160053680801430X PMC2961527 21202611

[bb9] Duque, J., Estevez-Hernandez, O., Reguera, E., Corrêa, R. S. & Gutierrez Maria, P. (2008). *Acta Cryst.* E**64**, o1068. 10.1107/S1600536808012208 PMC2961410 21202587

[bb10] Kemp, G., Roodt, A., Purcell, W. & Koch, K. R. (1997). *J. Chem. Soc. Dalton Trans.* **23**, 4481–4483.

[bb11] Koch, K. R., Sacht, C., Grimmbacher, T. & Bourne, S. (1995). *S. Afr. J. Chem.* **48**, 71–77.

[bb12] Mansuroğlu, D. S., Arslan, H., Flörke, U. & Külcü, N. (2008). *J. Coord. Chem.* **61**, 3134–3146.

[bb13] Nencki, M. (1873). *Ber. Dtsch. Chem. Ges.* **6**, 598–600.

[bb14] Özpozan, N., Arslan, H., Ozpozan, T., Ozdes, N. & Külcü, N. (2000). *Thermochim. Acta*, **343**, 127–133.

[bb22] Sheldrick, G. M. (2008). *Acta Cryst* A**64**, 112–122. 10.1107/S0108767307043930 18156677

[bb15] Thiam, E. I., Diop, M., Gaye, M., Sall, A. S. & Barry, A. H. (2008). *Acta Cryst.* E**64**, o776. 10.1107/S1600536808008374 PMC2961338 21202269

[bb16] Tutughamiarso, M. & Bolte, M. (2007). *Acta Cryst.* E**63**, o4682.

[bb17] Uğur, D., Arslan, H. & Külcü, N. (2006). *Russ. J. Coord. Chem.* **32**, 669–675.

[bb18] Xian, L. (2008). *Acta Cryst.* E**64**, o1969. 10.1107/S1600536808029425 PMC2959244 21201169

[bb19] Yue, H., Wang, Y., Xia, A., Luo, S. & Xu, D. (2008). *Acta Cryst.* E**64**, o858. 10.1107/S1600536808009768 PMC2961226 21202345

[bb20] Yusof, M. S. M., Ayob, N. A. C., Kadir, M. A. & Yamin, B. M. (2008*a*). *Acta Cryst.* E**64**, o937. 10.1107/S1600536808011495 PMC2961203 21202418

[bb21] Yusof, M. S. M., Muharam, S. H., Kassim, M. B. & Yamin, B. M. (2008*b*). *Acta Cryst.* E**64**, o1137. 10.1107/S1600536808014530 PMC2961577 21202646

